# A Study on the Effects of Finerenone on Hemoglobin, Hematocrit, and Serum Albumin Levels in Real-World Clinical Practice

**DOI:** 10.7759/cureus.110372

**Published:** 2026-06-06

**Authors:** Nakai Kazuaki

**Affiliations:** 1 Department of Internal Medicine, Ehime Rosai Hospital, Niihama, JPN

**Keywords:** finerenone, hematocrit, hemoglobin, serum albumin, trend shift analysis

## Abstract

Background

Large-scale clinical studies have shown that finerenone reduces the risk of cardiovascular and renal events in patients with type 2 diabetes and chronic kidney disease (CKD). Similarly, sodium-glucose cotransporter 2 inhibitors (SGLT2is) reduce the risk of cardiovascular death and heart failure in patients with type 2 diabetes at high cardiovascular risk, with contributing factors reported to include improvements in anemia and changes in plasma volume markers, such as serum albumin (ALB) levels. The impact of finerenone on anemia and plasma volume markers has not been reported. Therefore, we hypothesized that finerenone administration might improve these factors and examined the effects of its administration on these factors.

Materials and methods

This retrospective observational study included 20 patients with type 2 diabetes and CKD at our hospital who initiated finerenone and had no additional medications added during the 12 months before and after administration. Changes in clinical test data, including hemoglobin (Hb), hematocrit (Ht), and serum ALB levels, were evaluated using trend-shift analysis with a mixed-effects model. Repeated measurements before and after finerenone administration were collected, and the rate of change during each period was approximated with a linear trend line. A shift in the slope of the trend line was considered an intervention effect of finerenone. Analyses were performed for all 20 participants and were stratified by concomitant SGLT2i use and baseline anemia status.

Results

Overall, finerenone administration significantly increased Hb and Ht levels; however, the increase was significant only in the eight patients also taking SGLT2is and not in the 12 patients who were not. Among the five patients with anemia, Hb and Ht levels increased significantly, but no significant change was observed in the three anemic patients not receiving SGLT2is. Additionally, six patients receiving SGLT2is without anemia also showed significant increases in Hb and Ht levels. These findings suggest that the combination of finerenone and SGLT2is may improve anemia. Furthermore, serum ALB levels were significantly increased with combination therapy.

Conclusions

Our study suggests that finerenone may improve anemia and increase ALB levels when used in combination with SGLT2is. Since the cardiovascular death and heart failure risk reduction effects of SGLT2is have already been reported, it suggests that finerenone may act additionally to the effects of SGLT2is. We believe that adding finerenone to SGLT2is could potentially provide further cardiovascular and renal risk reduction effects.

## Introduction

Finerenone, a nonsteroidal mineralocorticoid receptor antagonist, has been reported to reduce the risk of cardiovascular and renal events in patients with type 2 diabetes and chronic kidney disease (CKD) [1-3]. Sodium-glucose cotransporter 2 inhibitors (SGLT2is) have also been shown to reduce the risk of cardiovascular death and heart failure in patients with type 2 diabetes with a history of cardiovascular disease or at high cardiovascular risk, with contributing factors reported to include increases in hemoglobin (Hb) and hematocrit (Ht) levels [4,5].

Anemia has been recognized as a factor that exacerbates the progression of cardiovascular disease, diabetic kidney disease, and diabetic retinopathy. In patients with type 2 diabetes who have a history of cardiovascular disease or are at high risk for it, correction of anemia has been associated with reduced cardiovascular mortality and prevention of heart failure [6]. Despite this, anemia often receives insufficient attention in routine clinical practice.

Finerenone’s impact on anemia has also not been evaluated. Therefore, we hypothesized that finerenone administration might improve anemia and investigated its effects on Hb and Ht levels in outpatients at our hospital. Additionally, since markers of plasma volume, such as albumin (ALB), uric acid, and lipid levels, may contribute to the risk-reducing effects of SGLT2is, we also examined the effects of finerenone on these parameters.

## Materials and methods

Study design

We conducted a retrospective real-world study at Ehime Rosai Hospital. The subjects were outpatients with type 2 diabetes and CKD at our hospital who were newly prescribed finerenone due to persistent proteinuria or low estimated glomerular filtration rate (eGFR) and who continuously received doses of 10 or 20 mg. The study period was approximately two years (i.e., 12-14 months before and 12-14 months after the initiation of finerenone administration). Since seasonal variation in HbA1c and eGFR has been reported [7,8], we speculated that confounding due to seasonal variation could be excluded by comparing values within the same season.

The primary outcome was the effect of finerenone administration on Hb and Ht levels, evaluated by comparing changes over time during the 12-14 months before and the 12-14 months after starting finerenone (study period). In addition, a sub-analysis was conducted by stratifying patients based on the presence or absence of concomitant SGLT2is and baseline anemia status at the start of finerenone administration.

Secondary evaluation items included chronological changes in parameters other than Hb and Ht levels during the study period, such as serum ALB, eGFR, serum uric acid, lipid levels, BMI, and blood pressure (BP). In some cases, changes in urinary ALB-to-creatinine ratio (UACR) were also assessed. Additionally, a subgroup analysis was conducted based on whether SGLT2is were co-administered at the initiation of finerenone.

All blood samples were drawn in the postprandial state during the study period. This study was conducted in accordance with the Declaration of Helsinki and was approved by the Ehime Rosai Hospital Clinical Trial Ethics Review Committee (approval number 116).

Inclusion and exclusion criteria

We identified 58 outpatients who were newly prescribed and continuously received finerenone at doses of 10 or 20 mg due to the aforementioned reasons. Among these 58 outpatients, we selected 20 outpatients who had no additional medications other than finerenone but had discontinued or reduced their diabetic concomitant medications (i.e., discontinuing metformin or reducing insulin dosage by 4-8 units during the study period).

We excluded 38 outpatients due to one of the following reasons: receiving an additional prescription of new agents during the study period; suspected kidney disease other than CKD; new onset of systemic disease; or laboratory data measured fewer than 10 times during the study period.

Data collection

Demographic and laboratory data for these 20 patients were collected from their medical records during the study period. The collected data included sex, age, BMI, systolic and diastolic BP, duration of diabetes, smoking habit, history of retinopathy and cardiovascular disease, concomitant medications, serum Hb, Ht, serum ALB, eGFR, urinary UACR, HbA1c, uric acid, total cholesterol, low-density lipoprotein cholesterol, high-density lipoprotein cholesterol (HDL-C), and non-HDL-C.

Because UACR values were not normally distributed and showed considerable variability, log-transformed values (log-UACR) were used for analysis. eGFR was calculated using the formula established by the working group of the Japanese CKD Initiative [9]:

$\mathrm{eGFR} \, (\mathrm{mL/min/1.73\,m^2}) = 194 \times \mathrm{Age}^{-0.287} \times \mathrm{Serum\ Creatinine}^{-1.094} \times 0.739 \; (\mathrm{if\ female})$

Non-HDL-C levels were calculated by subtracting HDL-C from total cholesterol. BMI was defined as body weight (kg) divided by height squared (m²). Smoking habit was defined as prior or current tobacco use.

Patients with missing UACR measurements in the medical records during the study periods were included if sufficient other laboratory data were available (≥5 measurements in both pre- and post-initiation of finerenone periods). All laboratory data repeatedly measured during the study period were recorded for each patient.

Statistical analysis

Results are presented as medians (first and third quartiles) or numbers (percentages). Because the primary evaluation items, Hb and Ht levels, were measured repeatedly, all repeated measurement data were collected, and trend-shift analysis (TSA) was performed using a mixed-effects model (MEM). The rate of change in each variable before and after finerenone administration was approximated by a linear trend line. The effect of finerenone was evaluated based on whether a shift in the slope of this line was observed. TSA was performed using MEMs with random intercepts.

Fixed effects included month (month), finerenone administration (Fine), and the interaction between month and finerenone administration (month: Fine). The random effect was set as differences in baseline laboratory values for each patient at finerenone initiation (random intercepts model). Specifically, the following formula was used:

Outcome ~ month + Fine + month:Fine + (1 | id)

A significant interaction between month and finerenone administration (Month:Fine; p < 0.05) was interpreted as a trend shift, indicating an intervention effect of finerenone. The same TSA approach was applied to secondary evaluation items using an MEM with random intercepts. All statistical analyses were performed with EZR (Jichi Medical University, Tochigi, Japan), which is a graphical user interface for R (The R Foundation for Statistical Computing, Vienna, Austria).

## Results

Patient characteristics

Table 1 shows the patient demographics and laboratory data at finerenone initiation. However, the dosage was adjusted according to precautions and recommended guidelines; therefore, the final dosage at the end of the study period and the number of patients at each dosage are also presented. Specifically, patients with an eGFR ≥60 mL/min/1.73 m² started at 20 mg/day, whereas those with an eGFR <60 mL/min/1.73 m² started at 10 mg/day. One month later, if the measured serum potassium level was ≤4.8 mmol/L and the decrease in eGFR was ≤30% compared with the previous value, the dose was increased to 20 mg. No patients required a treatment pause due to potassium levels exceeding 5.5 mmol/L.

**Table 1:** Demographics and laboratory data at finerenone initiation, including finerenone dosage and the number of patients at the final dosage ALB, albumin; ARB/ACE, angiotensin II receptor blocker/ACE inhibitor; CCB, calcium channel blocker; D-BP, diastolic blood pressure; DPP4i, dipeptidyl peptidase-4 inhibitor; EPO, erythropoietin; EPA, eicosapentaenoic acid; eGFR, estimated glomerular filtration rate; GLP-1RA, glucagon-like peptide-1 receptor agonist; Hb, hemoglobin; HbA1c, hemoglobin A1c; Ht, hematocrit; LDL-C, low-density lipoprotein cholesterol; Log-UACR, log-transformed urinary albumin-to-creatinine ratio; Met, metformin; non-HDL-C, non-high-density lipoprotein cholesterol; PPI, proton pump inhibitor; S-BP, systolic blood pressure; SGLT2i, sodium-glucose cotransporter 2 inhibitor; TG, triglyceride; UA, uric acid; UACR, urinary albumin-to-creatinine ratio; αGI, alpha-glucosidase inhibitor

Clinical and laboratory parameters	All patients (n = 20)	Co-administration of SGLT2is (n = 8)	With anemia (n = 5)
Gender (male/female), n	14/6	7/1	4/1
Age (years)	75 (69.8-80)	69.5 (63-75)	80 (75-81)
Disease duration (years)	18.5 (9.8-23)	21.5 (13.3-23)	20 (15-23)
Dose (20 mg/10 mg), n	11/9	6/2	3/2
Smoking habit, n (%)	4 (20.0)	2 (25.0)	1 (20.0)
Retinopathy, n (%)	3 (15.0)	1 (12.5)	1 (20.0)
BMI (kg/m²)	22.1 (21.2-23.4)	21.1 (20.7-23.3)	21.8 (21.5-23.2)
S-BP (mmHg)	126 (118-130)	125.5 (119.8-126.8)	127 (126-130)
D-BP (mmHg)	67 (62-79)	71 (63.3-78)	62 (61-62)
UACR (mg/g creatinine; n = 14)	53.7 (22.0-153.9)	215.7 (119.1-411.6; n = 4)	293.3 (165.9-529.9; n = 3)
Log-UACR (mg/g creatinine; n = 14)	4.0 (3.1-5.0)	5.3 (4.7-5.9; n = 4)	5.7 (4.7-6.2; n = 3)
eGFR (mL/min/1.73 m²)	49.6 (42.8-60.7)	52.1 (49.4-86.5)	42.9 (37.6-53.3)
UA (mg/dL)	5.4 (4.2-6.3)	4.2 (3.5-6.0)	6.0 (5.2-6.5)
ALB (g/dL)	4.3 (4.2-4.5)	4.4 (4.2-4.6)	4.2 (4.2-4.3)
Hb (g/dL)	13.7 (12.3-14.6)	14.6 (13.7-14.6)	11.9 (11-12.4)
Ht (%)	40.6 (37.1-42.6)	42.3 (41.0-43.3)	33.5 (32.5-37.4)
HbA1c (%)	6.5 (6.2-7.4)	6.7 (6.6-7.4)	6.4 (6.2-7.5)
TG (mg/dL)	95.5 (70.3-128.3)	95.5 (86-128.3)	109 (86-126)
LDL-C (mg/dL)	79 (60.5-87.8)	78 (68.8-82.5)	83 (59-95)
Non-HDL-C (mg/dL)	97 (81.5-112.3)	89 (82.3-101)	87 (83-113)
EPO use, n (%)	1 (5.0)	0 (0.0)	0 (0.0)
Statin use, n (%)	16 (80.0)	5 (62.5)	3 (60.0)
Ezetimibe use, n (%)	2 (10.0)	2 (25.0)	0 (0.0)
EPA use, n (%)	7 (35.0)	3 (37.5)	2 (40.0)
ARB/ACE inhibitor use, n (%)	9 (45.0)	4 (50.0)	2 (40.0)
CCB use, n (%)	18 (90.0)	6 (75.0)	4 (80.0)
Other antihypertensive agent use, n (%)	9 (45.0)	5 (62.5)	1 (20.0)
Allopurinol use, n (%)	4 (20.0)	2 (25.0)	2 (40.0)
PPI use, n (%)	6 (30.0)	2 (25.0)	1 (20.0)
Antiplatelet agent use, n (%)	8 (40.0)	2 (25.0)	1 (20.0)
SGLT2i use, n (%)	8 (40.0)	8 (100.0)	2 (40.0)
Metformin use, n (%)	8 (40.0)	4 (50.0)	3 (60.0)
DPP4i use, n (%)	9 (45.0)	2 (25.0)	4 (80.0)
GLP-1RA use, n (%)	6 (30.0)	4 (50.0)	1 (20.0)
Sulfonylurea or glinide use, n (%)	3 (15.0)	2 (25.0)	1 (20.0)
Insulin use, n (%)	7 (35.0)	2 (25.0)	1 (20.0)
αGI use, n (%)	2 (10.0)	0 (0.0)	1 (20.0)
Thiazolidinedione use, n (%)	0 (0.0)	0 (0.0)	0 (0.0)

Eight patients were administered SGLT2is. Anemia was defined as Hb levels <13 g/dL in men and <12 g/dL in women; five patients met this criterion, including two receiving SGLT2is. Patients exhibited a range from normal to overt albuminuria, but all cases with SGLT2i co-administration or anemia had at least microalbuminuria. All patients had eGFR ≥ G3 according to the CKD classification, with only one patient exceeding 90 mL/min/1.73 m². Serum ALB levels were ≥3.6 g/dL in all patients, within the normal range (3.5-5.0 g/dL). Calcium channel blockers were the most commonly prescribed concomitant medications. One patient was receiving darbepoetin alfa with no dosage changes during the study period.

Primary outcome

**Figure 1:**
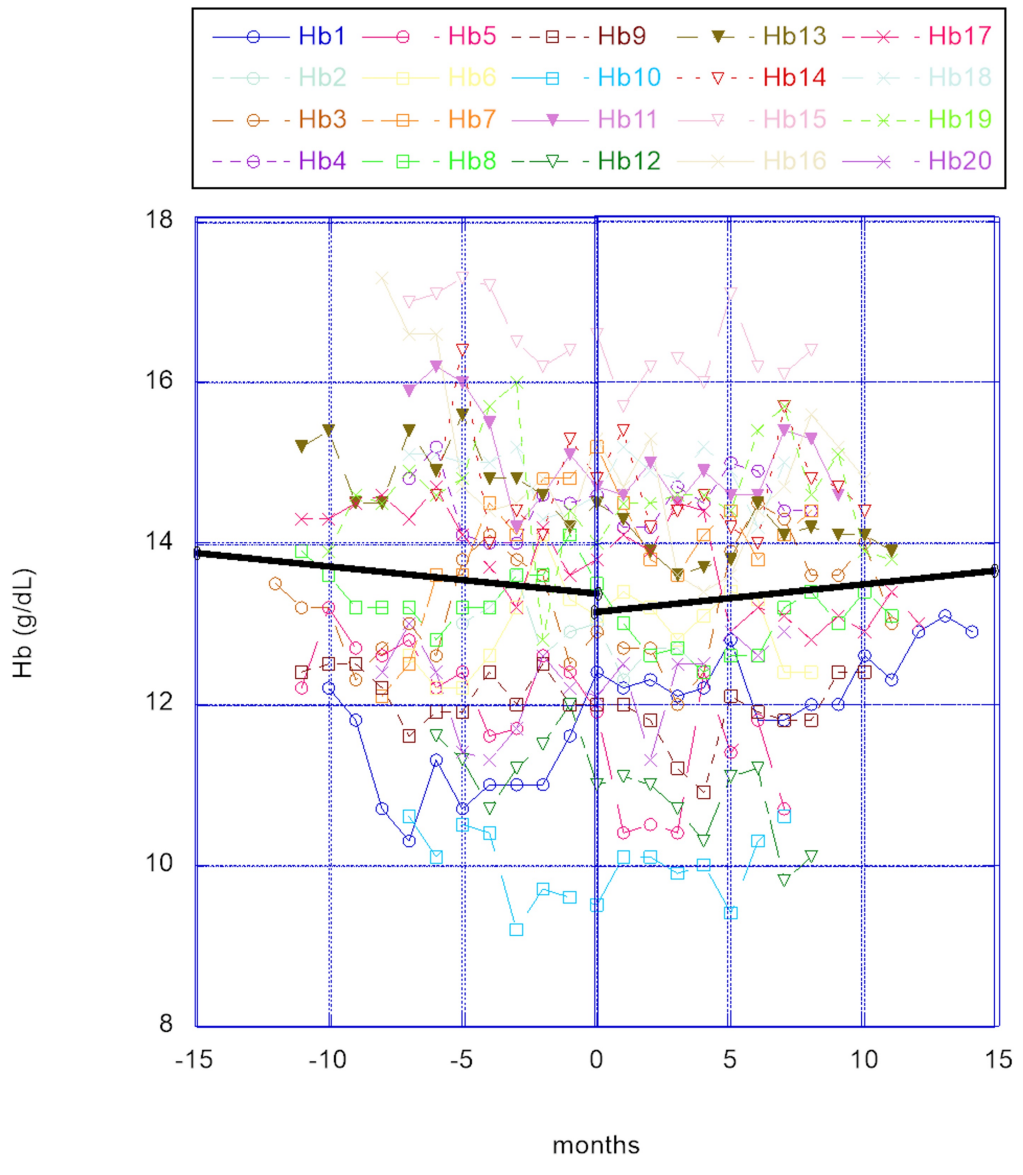
Changes in Hb levels over time in each of the 20 patients and their approximated linear trend lines Hb with a number indicates each patient’s value. The pre-initiation period of finerenone was denoted by a negative number. Hb, hemoglobin This image was created using KaleidaGraph version 5.04 (Synergy Software, Reading, PA, USA).

MEM analysis showed a significant increase in Hb levels following finerenone administration (Table 2), indicating a trend shift from a negative to a positive slope. Specifically, the “Fixed effects” column in Table 2 shows a p-value of 0.00199 for the interaction between month and finerenone administration (“month: Fine”), with an estimate of 0.06756. This indicates that Hb levels decreased by 0.03398 over time (numeric value corresponding to the “Estimate of month,” as described in the result column of the “Fixed effects”) during the pre-initiation period but increased by 0.03358 over time (the sum of the value corresponding to the “Estimate of month” (−0.03398) and the value corresponding to the “Estimate of month: Fine” (0.06756) described in the result column of the “Fixed effect”) during the post-initiation period. Figure 1 illustrates the approximate linear trends of the numerical changes before and after finerenone administration, and Table 3 presents the slopes.

**Table 2:** Results of TSA of Hb levels in all 20 patients ***p < 0.001, **p < 0.01, *p < 0.05 by MEMs with random intercepts Fine = finerenone administration; Month: Fine = the interaction between month and finerenone administration Hb, hemoglobin; MEM, mixed-effects model; TSA, trend-shift analysis

Random effects				
Group	Name	Variance	SD	
Id	(Intercept)	2.5527	1.5977	
Residual		0.3746	0.6121	
Number of observations: 373, groups: id, 20				
Fixed effects				
Term	Estimate	SD	t-value	p-value
(Intercept)	13.39474	0.36567	36.63	<2e-16***
Month	-0.03398	0.01452	-2.34	0.01985*
Fine	-0.24799	0.12049	-2.058	0.04031*
Month: Fine	0.06756	0.02169	3.115	0.00199**

**Table 3:** Results of TSA for Hb levels in the eight patients concurrently receiving SGLT2is ***p < 0.001, **p < 0.01, *p < 0.05 Fine = finerenone administration; Month: Fine = the interaction between month and finerenone administration Hb, hemoglobin; SGLT2i, sodium-glucose cotransporter 2 inhibitor; TSA, trend-shift analysis

Random effects				
Group	Name	Variance	SD	
id	(Intercept)	1.2516	1.1187	
Residual		0.4081	0.6389	
Number of observations: 156, groups: id, 8				
Fixed effects				
Term	Estimate	SE	t-value	p-value
(Intercept)	14.05522	0.415	33.868	4.32e-10***
Month	-0.06826	0.02183	-3.126	0.00214*
Fine	-0.33321	0.19916	-1.673	0.09646
Month: Fine	0.1214	0.0345	3.519	0.00058**

**Figure 2:**
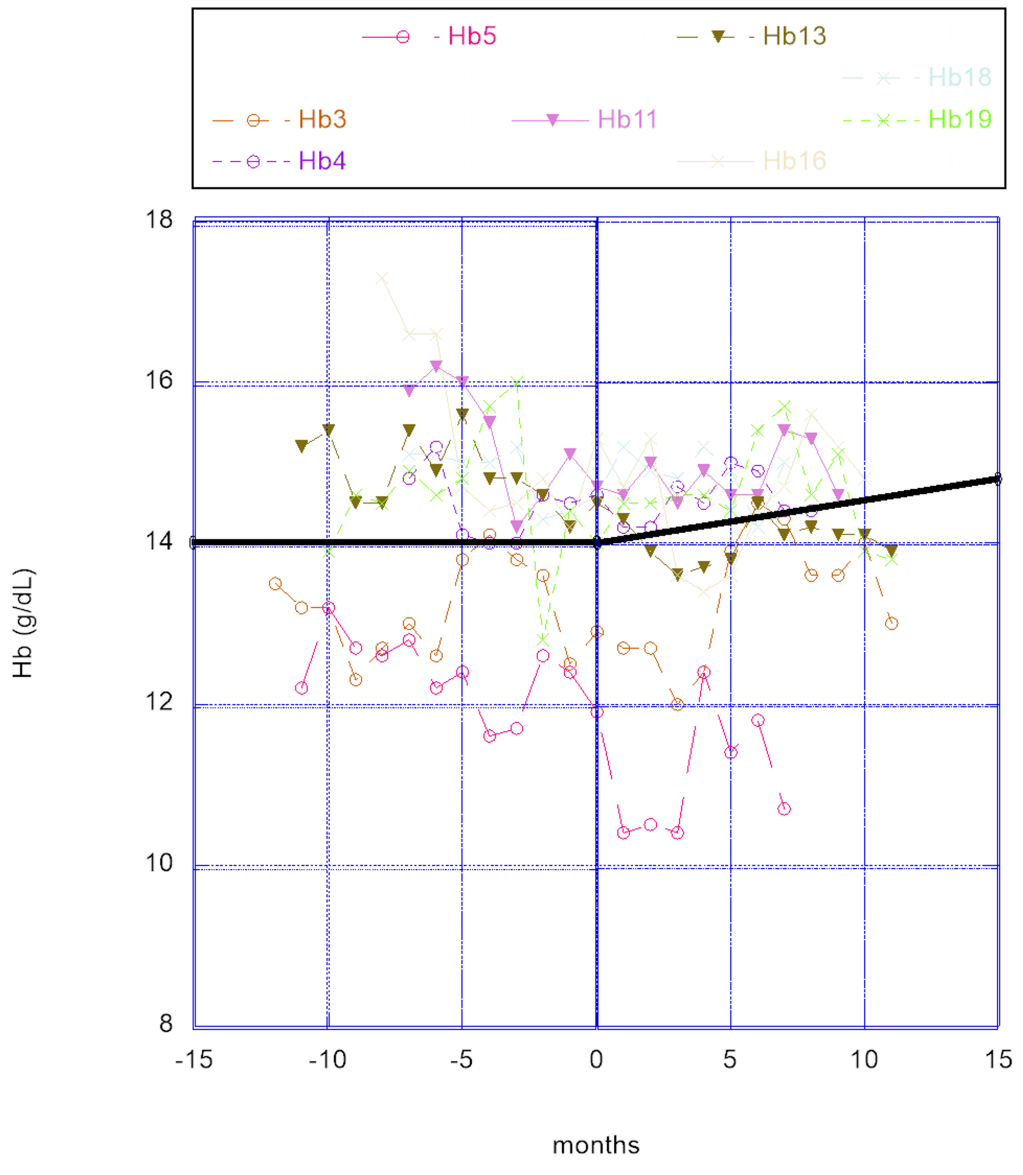
Changes in Hb levels over time in each of the eight patients receiving SGLT2is and their approximated linear trend lines Hb with a number indicates each patient’s value. The pre-initiation period of finerenone was denoted by a negative number. Hb, hemoglobin; SGLT2i, sodium-glucose cotransporter 2 inhibitor This image was created using KaleidaGraph version 5.04 (Synergy Software, Reading, PA, USA).

**Figure 3:**
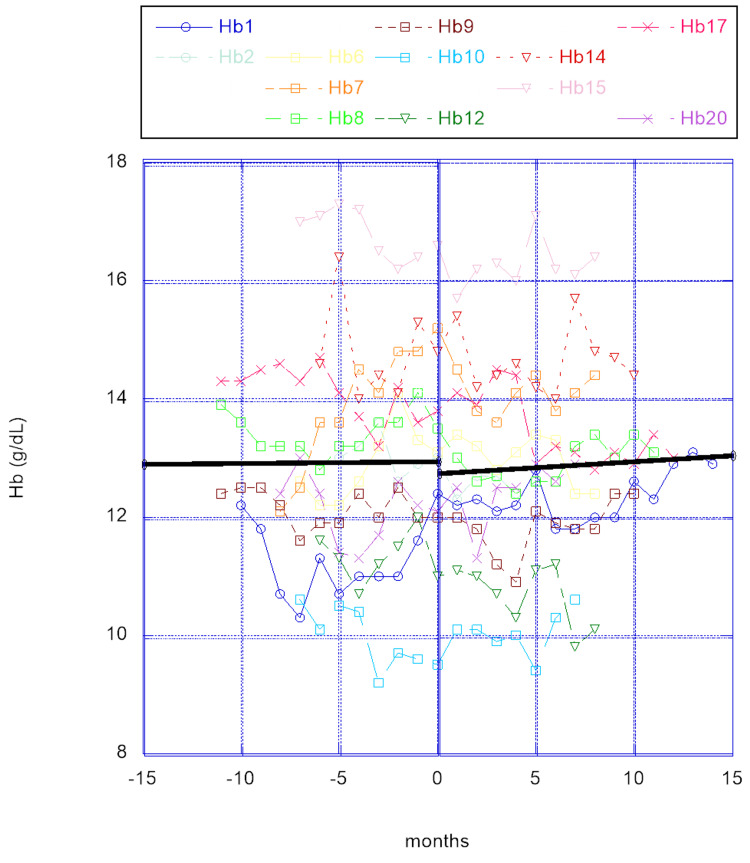
Changes in Hb levels over time in each of the 12 patients not receiving SGLT2is and their approximated linear trend lines Hb, hemoglobin; SGLT2i, sodium-glucose cotransporter 2 inhibitor This image was created using KaleidaGraph version 5.04 (Synergy Software, Reading, PA, USA).

In subgroup analysis, among the eight patients receiving SGLT2is, Hb levels shifted from an annual decrease of 0.06826 before finerenone to a significant annual increase of 0.05314 (Table 3, Table 4, Figure 2). The 12 patients not receiving SGLT2is did not show a significant increase (Table 4, Table 5, Figure 3). Among the five patients with anemia, Hb levels increased significantly, whereas the 15 without anemia did not show a significant increase (Table 4; results of TSA and graphs are not shown).

**Table 4:** p-value of the interaction between month and finerenone administration in the TSA for the primary evaluation items and the slope of the approximated linear trend lines before and after finerenone administration for the primary evaluation items ***p < 0.001, **p < 0.01, *p < 0.05 by MEMs with random intercepts Hb, hemoglobin; Ht, hematocrit; MEM, mixed-effects model; SGLT2i, sodium-glucose cotransporter 2 inhibitor; TSA, trend-shift analysis

Primary evaluation items	p-value	Before finerenone administration	After finerenone administration
All (n = 20)			
Hb	0.00199**	-0.03398	0.03358
Ht	0.0132*	-0.10221	0.05824
Those receiving SGLT2is (n = 8)			
Hb	0.00058***	-0.06826	0.05314
Ht	0.00348**	-0.19714	0.11343
Those not receiving SGLT2is (n = 12)			
Hb	0.524	0.002584	0.020145
Ht	0.766	-0.00246	0.02051
Those with anemia (n = 5)			
Hb	0.00134**	-0.02772	0.10898
Ht	0.00444**	-0.07535	0.26513
Those without anemia (n = 15)			
Hb	0.1193	-0.04025	-0.0135
Ht	0.0523	-0.12365	-0.2109
Those with anemia and receiving SGLT2is (n = 2)			
Hb	0.00367**	-0.02481	0.17771
Ht	0.00653**	-0.08686	0.44088
Those without anemia and receiving SGLT2is (n = 6)			
Hb	0.005977**	-0.10262	0.01041
Ht	0.02855*	-0.2799	0.00122
Those with anemia and not receiving SGLT2is (n = 3)			
Hb	0.33714	0.01501	0.07072
Ht	0.6	0.07636	0.16337
Those without anemia and not receiving SGLT2is (n = 9)			
Hb	0.65	0.001852	-0.012
Ht	0.564	-0.0167	-0.0695

**Table 5:** Results of TSA for Hb levels in the 12 patients not concurrently receiving SGLT2is ***p < 0.001 by MEMs with random intercepts Fine = finerenone administration; Month: Fine = the interaction between month and finerenone administration Hb, hemoglobin; MEM, mixed-effects model; SGLT2i, sodium-glucose cotransporter 2 inhibitor; TSA, trend-shift analysis

Random effects				
Group	Name	Variance	SD	
id	(Intercept)	2.9571	1.7196	
Residual		0.3358	0.5795	
Number of observations: 217, groups: id, 12				
Fixed effects				
Term	Estimate	SE	t-value	p-value
(Intercept)	12.99051	0.505887	25.679	1.19e-11***
Month	0.002584	0.019164	0.135	0.893
Fine	-0.22692	0.147524	-1.538	0.126
Month: Fine	0.017561	0.0275	0.639	0.524

Among the three anemic patients not receiving SGLT2is, no significant change in Hb levels was observed; conversely, among six non-anemic patients receiving SGLT2is, Hb levels increased significantly (Table 4; results of TSA and graphs are not shown).

**Figure 4:**
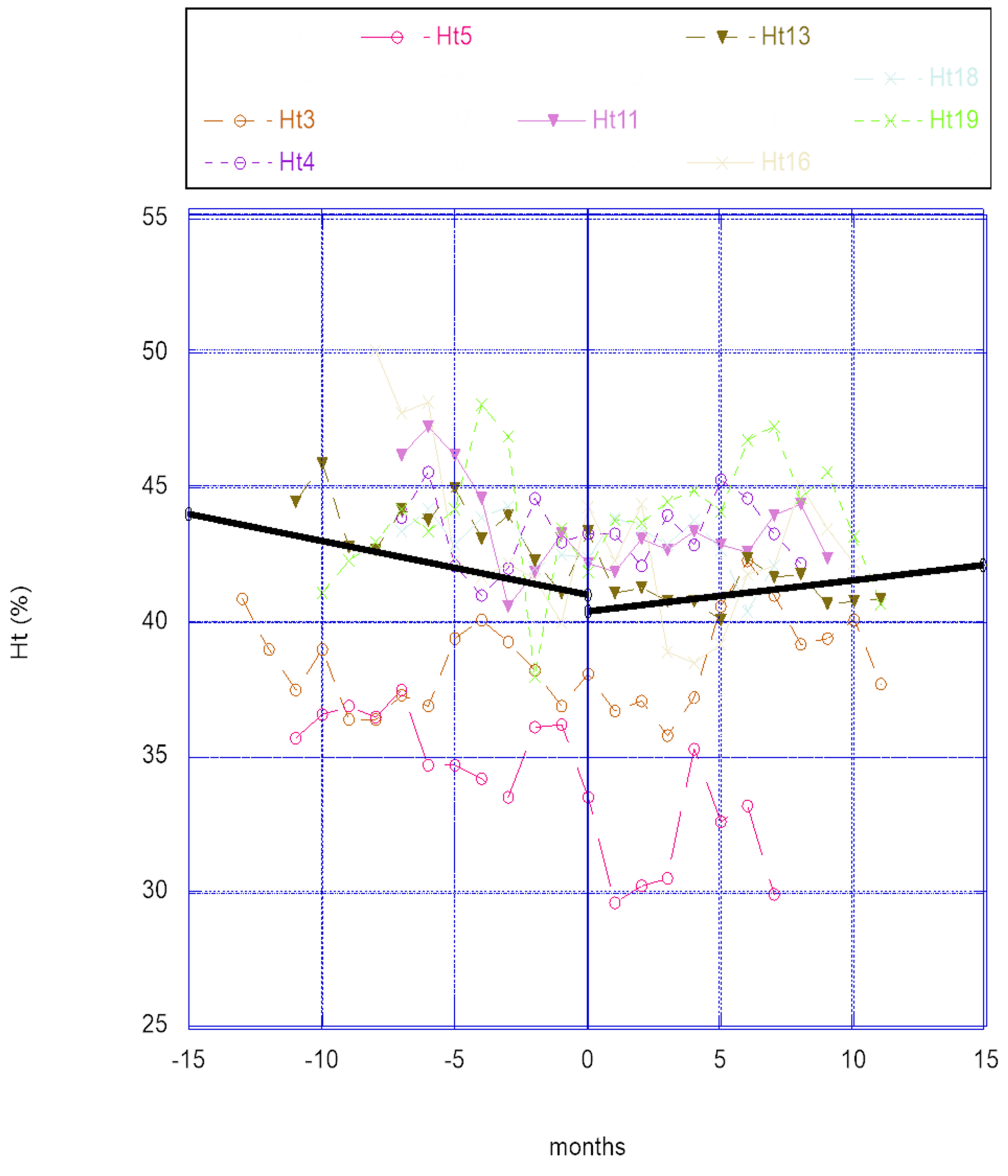
Changes in Ht levels over time in each of the eight patients receiving SGLT2is and their approximated linear trend lines Ht with a number indicates each patient’s value. The pre-initiation period of finerenone was denoted by a negative number. Ht, hematocrit; SGLT2i, sodium-glucose cotransporter 2 inhibitor This image was created using KaleidaGraph version 5.04 (Synergy Software, Reading, PA, USA).

**Figure 5:**
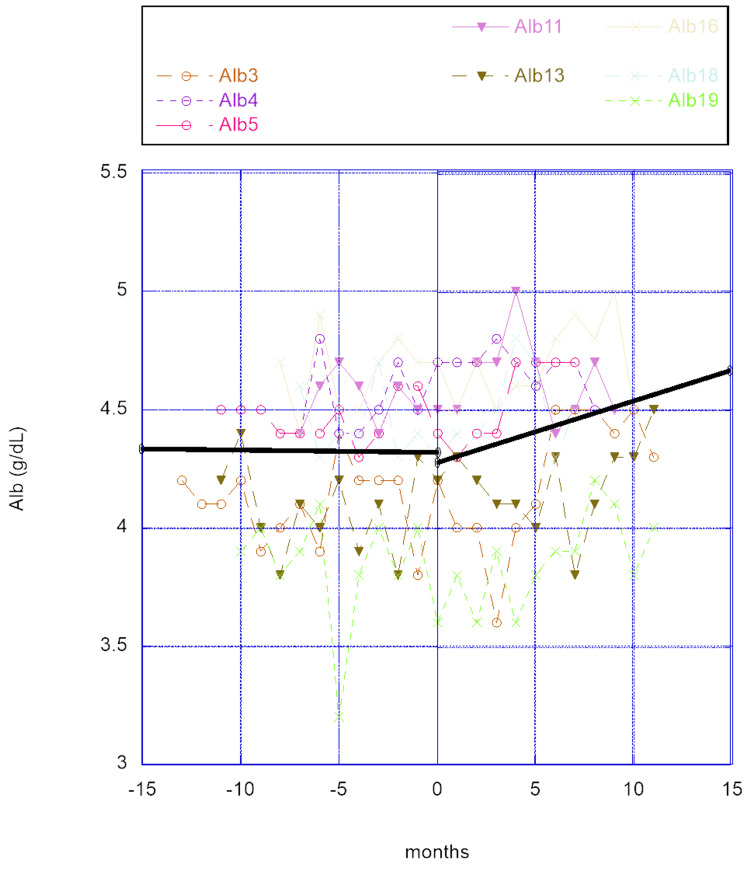
Changes in ALB levels over time in each of the eight patients receiving SGLT2is and their approximated linear trend lines ALB with a number indicates each patient’s value. The pre-initiation period of finerenone was denoted by a negative number. ALB, albumin; SGLT2i, sodium-glucose cotransporter 2 inhibitor This image was created using KaleidaGraph version 5.04 (Synergy Software, Reading, PA, USA).

Results for Ht levels mirrored those for Hb levels. In patients receiving SGLT2is, the annual rate shifted from a decrease of 0.19714 per year before finerenone administration to an increase of 0.11343 per year after initiation (Table 4, Table 6, Figure 4). Among secondary evaluation items, ALB levels showed a significant increase in MEM in the eight patients receiving concomitant SGLT2is, shifting from an annual decrease of 0.001437 before the start of finerenone to an annual increase of 0.02553 after its initiation (Table 7, Table 8, Figure 5). No significant changes were observed in the other secondary evaluation items.

**Table 6:** Results of TSA for Ht levels in the eight patients concurrently receiving SGLT2is ***p < 0.001, **p < 0.01 by MEMs with random intercepts Fine = finerenone administration; Month: Fine = the interaction between month and finerenone administration Ht, hematocrit; MEM, mixed-effects model; SGLT2i, sodium-glucose cotransporter 2 inhibitor; TSA, trend-shift analysis

Random effects				
Group	Name	Variance	SD	
id	(Intercept)	12.423	3.525	
Residual		3.747	1.936	
Number of observations: 156, groups: id, 8				
Fixed effects				
Term	Estimate	SE	t-value	p-value
(Intercept)	40.98789	1.30294	31.458	3.33e-10***
Month	-0.19714	0.06617	-2.98	0.00338**
Fine	-0.62054	0.60348	-1.028	0.30553
Month: Fine	0.31057	0.10455	2.97	0.00348**

**Table 7:** p-value of the interaction between month and finerenone administration in the TSA for the secondary evaluation items and the slope of the approximated linear trend lines before and after finerenone administration for the secondary evaluation items **p < 0.01 by MEMs with random intercepts ALB, albumin; D-BP, diastolic blood pressure; eGFR, estimated glomerular filtration rate; HbA1c, hemoglobin A1c; LDL-C, low-density lipoprotein cholesterol; MEM, mixed-effects model; non-HDL-C, non-high-density lipoprotein cholesterol; S-BP, systolic blood pressure; SGLT2i, sodium-glucose cotransporter 2 inhibitor; TSA, trend-shift analysis; UA, uric acid; UACR, urinary albumin-to-creatinine ratio

Secondary evaluation items	p-value	Before finerenone administration	After finerenone administration
All (n = 20)			
ALB	0.225	0.004655	0.012125
BMI	0.133	-0.06323	-0.03237
S-BP	0.616	-0.07546	0.08368
D-BP	0.42	-0.3457	0.0806
log UACR (n = 14)	0.301	-0.02088	-0.01753
eGFR	0.899	-0.11484	-0.13637
UA	0.8787	-0.02206	-0.01925
HbA1c	0.9884	-0.04538	-0.04507
LDL-C	0.395	0.2177	-0.1135
non-HDL-C	0.973	0.1043	0.11877
Those receiving SGLT2is (n = 8)			
ALB	0.00419**	-0.00144	0.02553
BMI	0.086	-0.06208	-0.00784
S-BP	0.189	-0.4968	0.2672
D-BP	0.081	-0.5824	0.2266
log UACR (n = 4)	0.514	0.01226	0.04619
eGFR	0.652	0.07792	0.20989
UA	0.482	-0.03267	-0.01526
HbA1c	0.115	-0.00856	-0.04347
LDL-C	0.695	-0.2091	0.0543
Non-HDL-C	0.196	-0.7425	0.183

**Table 8:** Results of TSA for ALB levels in the eight patients concurrently receiving SGLT2is ***p < 0.001, **p < 0.01 by MEMs with random intercepts Fine = finerenone administration; Month: Fine = the interaction between month and finerenone administration MEM, mixed-effects model; SGLT2i, sodium-glucose cotransporter 2 inhibitor; TSA, trend-shift analysis

Random effects				
Group	Name	Variance	SD	
Id	(Intercept)	0.0881	0.2968	
Residual		0.02946	0.1716	
Number of observations: 156, groups: id, 8				
Fixed effects				
Term	Estimate	SE	t-value	p-value
(Intercept)	4.323973	0.110234	39.226	1.18e-10***
Month	-0.00144	0.005866	-0.245	0.80683
Fine	-0.04042	0.053503	-0.755	0.45118
Month: Fine	0.02697	0.009269	2.91	0.00419**

## Discussion

In the 20 patients in this study, finerenone administration significantly increased Hb and Ht levels. However, subgroup analysis indicated that the increase occurred primarily in patients already using SGLT2is, suggesting that this effect may be due to combination therapy. Among secondary endpoints, ALB also showed a significant increase, further supporting a combined effect with SGLT2is.

The increase in Hb and Ht due to finerenone has previously only been reported in a post-hoc analysis of the FIDELITY trial [10]. In patients with baseline anemia (Hb <13 g/dL in men and <12 g/dL in women), Hb and Ht increased; however, a similar increase was observed in the placebo group, possibly because the anemic group had more advanced CKD and received higher doses of hematopoietic stimulants. Although not specific to finerenone, previous reports have shown that anemia caused by angiotensin II receptor blockers or angiotensin-converting enzyme inhibitors improved after switching to a mineralocorticoid receptor antagonist [11]. In this study, one patient was receiving a hematopoietic stimulant, but the dosage remained unchanged during the study period. There were also no changes in the use or dosage of angiotensin II receptor blockers or angiotensin-converting enzyme inhibitors. Subgroup analysis revealed no significant increases in Hb or Ht among the 15 patients without anemia, whereas significant increases were observed in the five patients with anemia, but only in the three patients who were also using SGLT2is. These results further suggest a combined effect with SGLT2is.

The mechanism by which SGLT2is increase Hb and Ht is thought to include hemoconcentration due to diuretic effects, hematopoietic stimulation via increased erythropoietin production, and anti-inflammatory, antioxidant, and antifibrotic effects through reduced glucose concentrations in the renal tubular interstitium. Suppression of hepcidin has also been reported [12]. Considering the significant increase in ALB observed with combination therapy, hemoconcentration is likely the primary mechanism in this study. An increase in ALB with finerenone has been reported in patients with nephrotic-range proteinuria receiving triple therapy with finerenone, renin-angiotensin system inhibitors, and dapagliflozin [13]. It was hypothesized that the reduction in proteinuria led to an increase in ALB levels. In our study, possibly due to the small sample size, a significant reduction in UACR was not observed. However, given the known anti-inflammatory effects of finerenone [14], it may have acted synergistically with SGLT2is.

In patients with type 2 diabetes and a history of cardiovascular disease or at high cardiovascular risk, improving anemia is thought to contribute to reduced cardiovascular death and prevention of heart failure. Conversely, in patients with advanced diabetes and established vascular complications, improving anemia may have adverse effects and increase mortality [6]. In this study, the addition of finerenone to SGLT2i therapy led to an improvement in Hb of 0.12 g/dL (from −0.07 to +0.05 g/dL) and an improvement in Ht of 0.3% (from −0.2 to +0.1). Compared with the improvements reported in the EMPA-REG OUTCOME study (approximately +0.8 g/dL in Hb and +5% in Ht), these changes are modest [15]. Nevertheless, careful observation is warranted to determine whether this modest improvement has any beneficial effects.

Limitations

This study has several limitations. First, it is a single-center study with a small sample size. Second, the study period was relatively short. Finally, we did not evaluate lifestyle factors, such as diet or physical activity, which may have influenced the findings. Therefore, definitive conclusions cannot be drawn. However, the study also has strengths. We analyzed all clinical data, including variations, using TSA. This approach allowed for evaluation more closely aligned with real clinical practice compared with methods assessing outcomes at only two time points before and after the intervention. Since each patient’s pre-finerenone period could serve as a control, confounding was minimized, allowing for a more accurate assessment of the effects of finerenone administration.

## Conclusions

In this study, adding finerenone to patients already taking SGLT2 inhibitors was associated with an improvement in anemia, likely influenced by hemoconcentration. Although improvements in anemia are associated with reduced cardiovascular death and prevention of heart failure, the effects of finerenone on anemia have rarely been reported. Further investigation is warranted to determine whether the reduction in cardiovascular and renal events with finerenone includes improvement in anemia.
